# Proapoptotic and Antiproliferative Effects of *Thymus caramanicus* on Human Breast Cancer Cell Line (MCF-7) and Its Interaction with Anticancer Drug Vincristine

**DOI:** 10.1155/2014/893247

**Published:** 2014-04-09

**Authors:** Saeed Esmaeili-Mahani, Farzaneh Falahi, Mohammad Mehdi Yaghoobi

**Affiliations:** ^1^Laboratory of Molecular Neuroscience, Kerman Neuroscience Research Center, Institute of Neuropharmacology, Kerman University of Medical Sciences, Kerman, Iran; ^2^Department of Biology, Faculty of Sciences, Shahid Bahonar University of Kerman, P.O. Box 76135-133, Kerman, Iran; ^3^Research Department of Biotechnology, Institute of Science and High Technology and Environmental Sciences, Graduate University of Advanced Technology, Kerman, Iran

## Abstract

*Thymus caramanicus Jalas* is one of the species of thymus that grows in the wild in different regions of Iran. Traditionally, leaves of this plant are used in the treatment of diabetes, arthritis, and cancerous situation. Therefore, the present study was designed to investigate the selective cytotoxic and antiproliferative properties of *Thymus caramanicus* extract (TCE). MCF-7 human breast cancer cells were used in this study. Cytotoxicity of the extract was determined using MTT and neutral red assays. Biochemical markers of apoptosis (caspase 3, Bax, and Bcl-2) and cell proliferation (cyclin D1) were evaluated by immunoblotting. Vincristine was used as anticancer control drug in extract combination therapy. The data showed that incubation of cells with TCE (200 and 250 **μ**g/mL) significantly increased cell damage, activated caspase 3 and Bax/Bcl2 ratio. In addition, cyclin D1 was significantly decreased in TCE-treated cells. Furthermore, concomitant treatment of cells with extract and anticancer drug produced a significant cytotoxic effect as compared to extract or drugs alone. In conclusion, thymus extract has a potential proapoptotic/antiproliferative property against human breast cancer cells and its combination with chemotherapeutic agent vincristine may induce cell death effectively and be a potent modality to treat this type of cancer.

## 1. Introduction

Cancer is a fatal disease, so it is critical to find beneficial solution to defeat this public health menace. Almost all chemotherapy drugs used to treat various cancers have unwanted and serious side effects. The development of resistance to multiple drugs is a common clinical problem in the treatment of various cancers. In addition, common cytotoxic therapies primarily target rapidly dividing cells including malignant cells as well as certain normal cells, leading to significant morbidity and limited clinical benefits of troubled patients.

Breast cancer is common well known cancer and is the main death reason of cancer in woman throughout the world. According to latest statistics, there were 39510 deaths of women and 410 deaths of men in 2012 in united stated and also they estimated 226870 new cancer cases in women and 2190 in men [1]. Currently, it seems that there is an urgent need for improvements in detection, diagnosis, and treatment of breast cancer. Unfortunately, the current classical treatments (surgery, chemotherapy, and/or radiotherapy) are impeded by side effect most importantly development of tumor resistant, loss of appetite, nausea and vomiting, weakness and fatigue, mouth soreness, hair loss, weight gain, premature menopause, lowered resistance to infections, bleeding, and diarrhea [2].

Therefore, finding novel and effective therapies against breast cancer is a current scientific challenge. Increasing attention has been paid to naturally acquired compounds as new candidates [3]. The renewed interest in natural substances has focused attention on plants used as foods, vegetables, fruits, or spices, which are a rich source of bionutrients or bioactive phytochemical and more detailed studies are needed to find the safety of these compounds [4].* Thymus* genus, which belongs to Lamiaceae family, is among the most popular plants throughout the world, commonly used as herbal teas, flavoring agents (condiment and spice), and aromatic and medicinal plants [5]. Furthermore, the essential oil and extract of different* Thymus* species are widely used in pharmaceutical, cosmetic, and perfume industry, as well as for flavoring and preservation of several food products [6]. Leaves and aerial part of this genus have also been used as herbal tea, antiseptic, antitussive, carminative, and flavoring agents [7].* Thymus caramanicus Jalas* (Avishan Kermani in Persian) is one of the 400 species of* Thymus* that grows in the wild in different regions of Iran. In traditional folk medicine, leaves of this plant are used in the treatment of rheumatism and skin disorders and as an antibacterial agent [8]. The major constituent of essential oil and extract from the aerial parts of this plant are carvacrol, thymol, p-cymene, *γ*-terpinene, and borneol, respectively [9]. The beneficial health properties of thymol and carvacrol as main components of* T. caramanicus* have encouraged us to look into its anticancer activity. To our best knowledge, reports are not available on the anticancer activity of* T. caramanicus* on cancer cells. Therefore, the present study is an attempt towards exploring the potential anticancer activity of this plant and its interaction with anticancer drugs on MCF-7 human breast adenocarcinoma cell line.

## 2. Materials and Methods

### 2.1. Materials

Cell culture reagents, penicillin-streptomycin solution, trypsin EDTA, fetal bovine serum (FBS), and heat-inactivated horse serum (HS) were obtained from Biosera Co. (East Sussex, UK). Culture flasks and dishes were acquired from SPL Lifesciences Inc. (Gyeonggi-Do, South Korea). 3-[4,5-Dimethyl-2-thiazolyl]-2,5-diphenyl-2-tetrazolium bromide (MTT), neutral red, and vincristine were purchased from Sigma (St. Louis, MI, USA). Primary polyclonal anti-caspase 3 and primary monoclonal anti-*β*-actin antibodies were purchased from Cell Signaling Technology, Inc. (Beverly, MA, USA). Primary polyclonal anti-Bax, anti-cyclin D1, and primary monoclonal anti-Bcl-2 antibodies were obtained from Santa Cruz Biotechnology, Inc. (Delaware Ave. Santa Cruz, USA).

### 2.2. Plant Material and Preparation of Its Extract

The aerial parts of* T. caramanicus* were collected from Hezar Mountain (Kerman province, Southern part of Iran) at the flowering stagein June 2012. The voucher specimens were deposited at the Herbarium of the Shahid Bahonar University of Kerman (Kerman, Iran). Two hundred grams of the air-dried leaves of* T. caramanicus* were grinded into fine powder, and then the grinded powder was extracted with 1.5 litter of ethanol and water mixture solvent (80 : 20) for three times. The collective hydroethanolic extracted was filtered through a filter membrane. After filtration process, the crude extract was completely dried with adding sodium sulphate. The solvent extracted was evaporated in rotary evaporator. Gas chromatography-mass spectroscopy (GC-MS) analysis of the extract showed that carvacrol (51.0%), thymol (20.84%), borneol (6.80%), cymene (6.25%), gamma-terpinene (5.50%), and beta myrcene (1.63%) were some constituents of the extract. The extract was weighted and dissolved in PBS buffer and used freshly.

### 2.3. Cell Culture

MCF-7 (human breast adenocarcinoma cell line) cells were obtained from National Cell Bank of Iran (NCBI). MCF-7 cells were grown with Dulbecco's modified Eagle's medium supplemented with 10% fetal bovine serum, penicillin (100 U/mL), and streptomycin (100 g/mL). They were maintained at 37°C in a 5% CO_2_ atmosphere. Growth medium was changed three times a week. Cells were plated at the density of 5000 per well in a 96-microplate well for the MTT and Neutral Red assays. For protein extraction, cells were grown in a 6-plate well and permitted to attach and grow for 24 h. Then the cells were incubated with different concentration of the extract alone or in combination with anticancer drugs.

### 2.4. Cell Viability Analysis

#### 2.4.1. MTT Assay

Cellular viability was assessed by the reduction of 2-(4,5-dimethylthiazol-2-yl)-2,5-diphenyltetrazolium bromide (MTT) to formazan. MTT was dissolved in PBS and added to the culture at final concentration of 0.5 mg/mL. After additional 2 h incubation at 37°C, the media were carefully removed and 100 *μ*L DMSO was added to each well, and the absorbance (OD) values were determined by spectrophotometry at 490 nm with microplate reader (Eliza MAT 2000, DRG Instruments, GmbH). Each experiment was performed 5-6 independent times. Results were expressed as percentages of control.

#### 2.4.2. Neutral Red Assay

The neutral red assay has been used extensively for* in vitro* assessment of cytotoxicity of infectious agents, food additives, and pharmaceuticals. This assay is based on the incorporation of neutral red (3-amino-7dimethyl-1-2-methylphenazine hydrochloride) into the lysosomes of viable cells after being incubated with test agents. Neutral red (4 mg/mL) was diluted 1 : 100 into medium and incubated overnight at 37°C and centrifuged before use. 200 *μ*L of prepared neutral red solution was added to each well and the cells were incubated at 37°C for 3 h. After that the cells were rapidly washed with a solution of 1% calcium chloride and 0.5% formaldehyde. The dye is then extracted from the intact and viable cell with a solution of 1% acetic acid and 50% ethanol and after 10 min incubation in room temperature and absorbance (OD) values were measured by spectrophotometry at 540 nm. Results were expressed as percentages of control.

### 2.5. Immunoblot Analysis

MCF-7 cells were homogenized in ice-cold buffer containing 10 mM Tris-HCl (pH 7.4), 1 mM EDTA, 0.1% SDS, 0.1% Na-deoxycholate, 1% NP-40 with protease inhibitors (1 mM phenylmethylsulfonyl fluoride, 2.5 *μ*g/mL of leupeptin, 10 *μ*g/mL of aprotinin), and 1 mM sodium orthovanadate. The homogenate was centrifuged at 14000 g for 15 min at 4°C. The resulting supernatant was retained as the whole cell fraction. Protein concentrations were measured using the Bradford method and equal amounts of protein (40 *μ*g) were resolved electrophoretically on a 9% SDS-PAGE gel and then transferred to nitrocellulose membranes (Hybond ECL, GE Healthcare Bio-Sciences Corp. NJ, USA). After overnight blocking at 4°C with 5% nonfat dried milk in Tris-buffered saline with Tween 20 (blocking buffer, TBS-T, 150 mM NaCl, 20 mM Tris-HCl, pH 7.5, 0.1% Tween 20), the membranes were probed with rabbit monoclonal antibody to caspase 3 (Cell Signaling Technology, USA, 1 : 1000 overnight at 4°C), Bax (Δ21): sc-6236, Bcl-2 (C-2): sc-7382, cyclin D1 (H-295): sc-753 (Santa Cruz, USA, 1 : 1000) for three hours at room temperature. After washing in TBS-T (three times, each time 5 min), the blots were incubated for 60 min at room temperature with a horseradish peroxidase-conjugated secondary antibody (1 : 15000, GE Healthcare Bio-Sciences Corp. NJ, USA). All antibodies were diluted in blocking buffer. The antibody-antigen complexes were detected using the ECL system and exposed to Lumi-Film chemiluminescent detection film (Roch, Germany). Lab Work analyzing software (UVP, UK) was used to analyze the intensity of the expression. *β*-actin immunoblotting (antibody from Cell Signaling Technology, INC. Beverly, MA, USA; 1 : 1000) was used to control for loading. The immunoblot experiments for each protein were performed 3-4 independent times.

### 2.6. Statistical Analysis

The results are expressed as mean ± SEM. The differences in mean cell viability assays between experimental groups were determined by one-way ANOVA, followed by Tukey HSD test. The values of caspase 3, Bax, Bcl-2, cyclin D1, and *β*-actin band density were obtained from band densitometry. These values were expressed as tested proteins/*β*-actin ratio for each sample. The averages for different groups were compared by ANOVA, followed by Tukey test. *P* < 0.05 was considered significant.

## 3. Results

### 3.1. The Effects of* Thymus caramanicus* Extract (TCE) on Cell Viability

**Figure 1:**
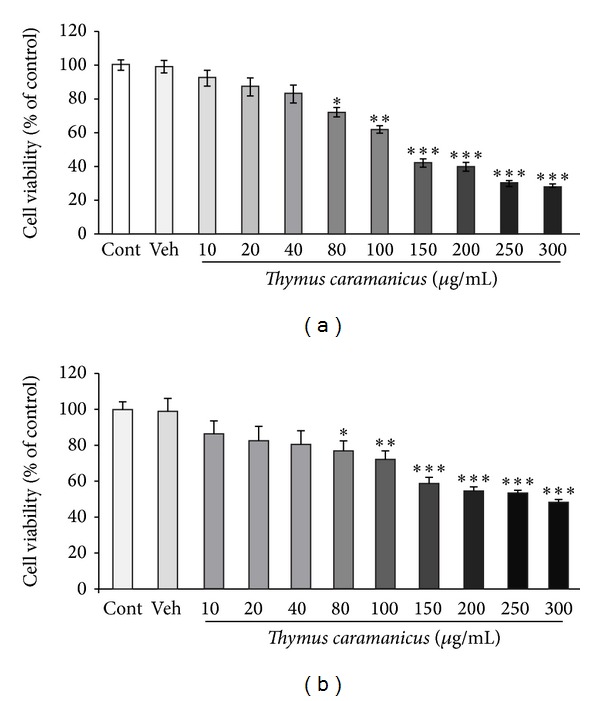
Effect of different doses of* T. caramanicus* extract (TCE) on MCF-7 cancer cells viability determined by MTT (a) and neutral red (b) assays. The extract has a dose dependent toxic effect on cancer cells. Data are expressed as mean ± SEM; *n* = 6 wells for each group; *P < 0.05, **P < 0.01, and ***P < 0.001 versus control nontreated cells.

At first, we analyzed the effects of different concentration of the extract on MCF-7 cell viability using the MTT and NR assays. After 24 h attachment/grow period, the cells were exposed to different concentrations of TCE (10, 20, 40, 80, 100, 150, 200, 250, and 300 *μ*g/mL) for a 24 h period.  Figure 1(a) shows that the extract could decrease cell viability in a dose dependent manner in this cancer cell line. Such cytotoxicity was also observed in neutral red assay (Figure 1(b)). TCE in doses of 150, 200, 250, and 300 *μ*g/mL potently elicited cell damage after 24 h and had a moderate effect in 80 and 100 *μ*g/mL, while it could not prevent cell damage in dose of 10, 20, and 40 *μ*g/mL.

### 3.2. The Effects of Noneffective and Subeffective Doses of* T. caramanicus* Extract (TCE) Alone or in Combination with Anticancer Drug Vincristine on Cell Viability

For combination therapy, we assessed the effect of non- and subeffective doses of TCE (40 and 80 *μ*g/mL, resp.) plus vincristine (as a common anticancer drug). Vincristine at doses of 150 nM did not show a significant toxic effect on MCF-7 cells. Therefore, this dose was used for combination therapy with* T. caramanicus* extract.

**Figure 2:**
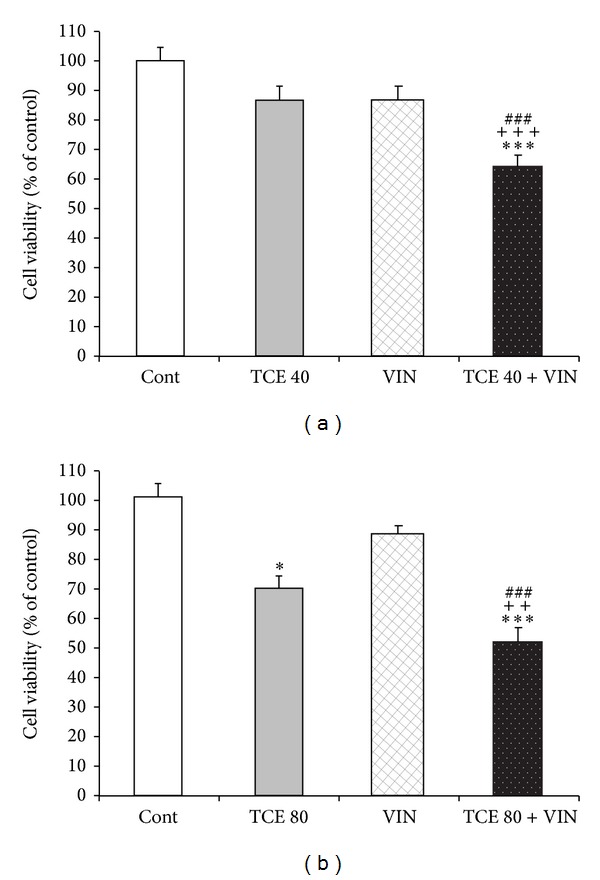
Effect of noneffective (40 *μ*g/mL) and subeffective (80 *μ*g/mL) doses of* T. caramanicus* extract (TCE) alone or in combination with 150 nM vincristine on MCF-7 cell viability determined by MTT assay. Data are expressed as mean ± SEM; *n* = 6 wells for each group; *P < 0.05 and ***P < 0.001 versus control cells. ^++^ *P* < 0.01 and ^+++^ *P* < 0.001 versus cells that had TCE alone. ^###^ *P* < 0.001 versus vincristine-treated cell.

As shown in Figure 2, TCE (40 and 80 *μ*g/mL) significantly potentiated the effect of vincristine. TCE (40 *μ*g/mL) did not have any toxic effect, but its concomitant treatment with a subeffective dose of vincristine produced significant cytotoxic effect (Figure 2(a)). Furthermore, 80 *μ*g/mL TCE in combination with vincristine had a potent cytotoxic effect which was greater than those observed in cells treated with TCE or vincristine alone (Figure 2(b)).

### 3.3. Western Blot Analysis of Cleaved Caspase 3, Bax, Bcl-2, and Cyclin D1 in MCF-7 Cells Treated with* T. caramanicus* Extract (TCE)

**Figure 3:**
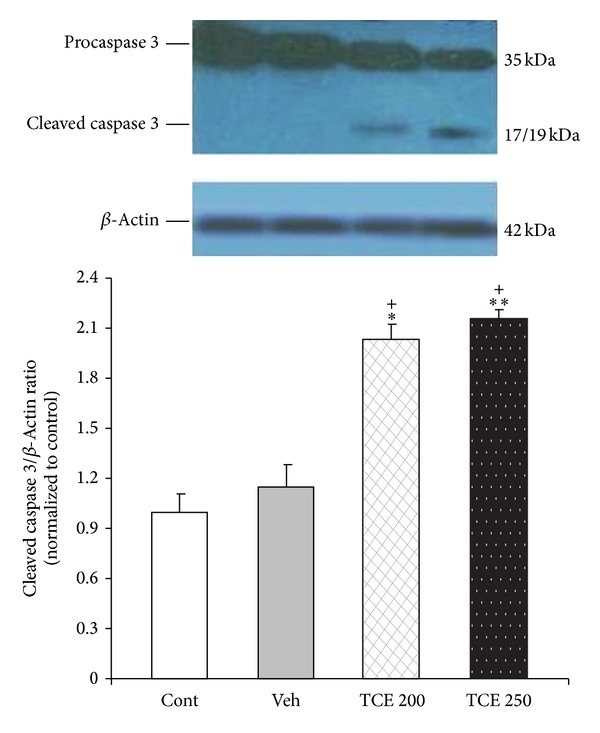
The activation of caspase 3 protein in MCF-7 cells exposed to 200 and 250 *μ*g/mL of* T. caramanicus* extract (TCE) for 24 h. Each value represents the mean ± SEM band density ratio for each group. *β*-actin was used as an internal control. *P < 0.05, **P < 0.01 significantly different versus control cells. ^+^ *P* < 0.001 versus vehicle-treated cells.

To examine the potential mediators of TCE-induced cell damage, we analyzed caspase 3 activation and Bax : Bcl-2 proteins ratio as cell apoptosis markers. The cells were exposed to 200 and 250 *μ*g/mL of TCE (the most effective doses in MTT and neutral red assays) for 24 h. The amount of cleaved caspase 3 in TCE-treated MCF-7 cells was found to be increased (*P* < 0.001) compared to that in the cells treated with control medium (Figure 3).

**Figure 4:**
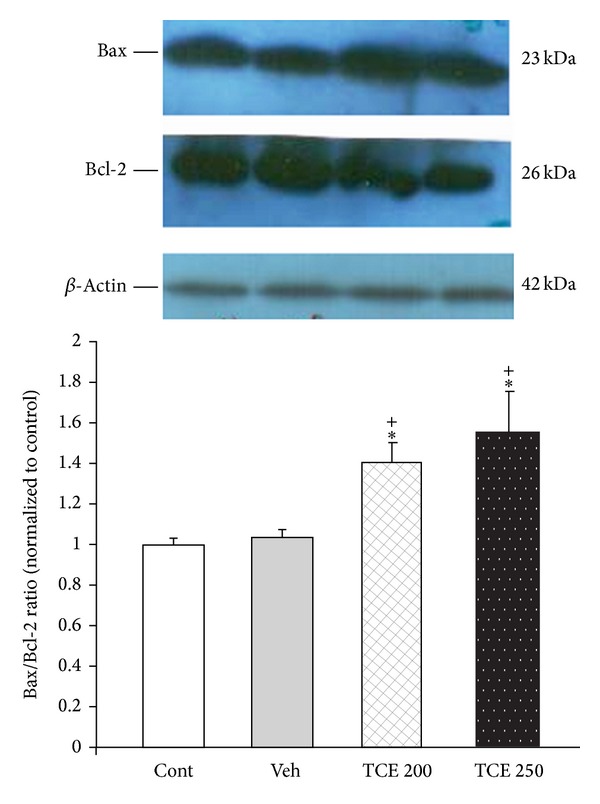
Effect of* T. caramanicus* extract (TCE) on the levels of Bax and Bcl-2 protein expression in MCF-7 cancer cell line. Bax and Bcl-2 protein levels were assayed by western blotting. ***P < 0.001 significantly different versus control cells. *P < 0.05 significantly different versus control cells. ^+^ *P* < 0.001 versus vehicle-treated cells.

Additionally, Bax protein was significantly increased in TCE-treated cells, while the Bcl-2 protein decreased. Consequently, there was a significant increase (*P* < 0.05) in the Bax : Bcl-2 protein ratio in the cells exposed to 200 and 250 *μ*g/mL of TCE (Figure 4).

**Figure 5:**
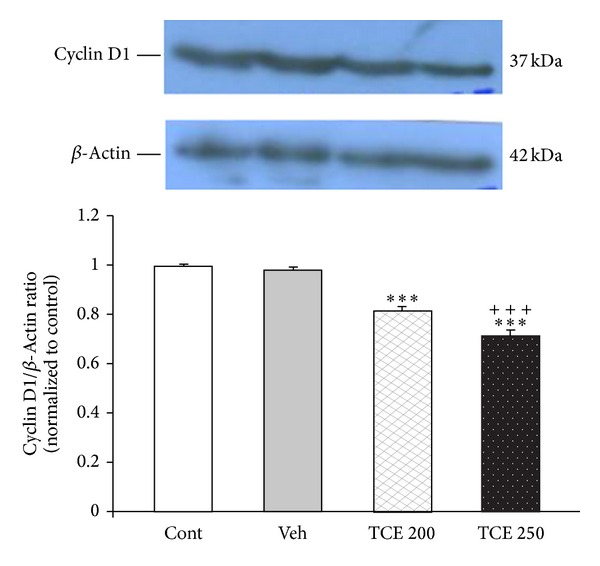
Effect of* T. caramanicus* extract (TCE) on the level of cyclin D1 in MCF-7 cells. Each value represents the mean ± SEM band density ratio for each group. *β*-actin was used as an internal control. ***P < 0.001 significantly different versus control cells. ^+^ *P* < 0.05, ^++^ *P* < 0.01 versus vehicle-treated cells.

As shown in Figure 5, there was a significant decrease in cyclin D1 protein level (as a marker of cell proliferation) in TCE-treated MCF-7 cells (*P* < 0.001).

These results indicate that TCE may disturb the balance of positive and negative regulators of apoptosis, resulting in increased cell death.

### 3.4. Western Blot Analysis of Cleaved Caspase 3 and Cyclin D1 in MCF-7 Cells Treated with* T. caramanicus* Extract (TCE) and Vincristine

**Figure 6:**
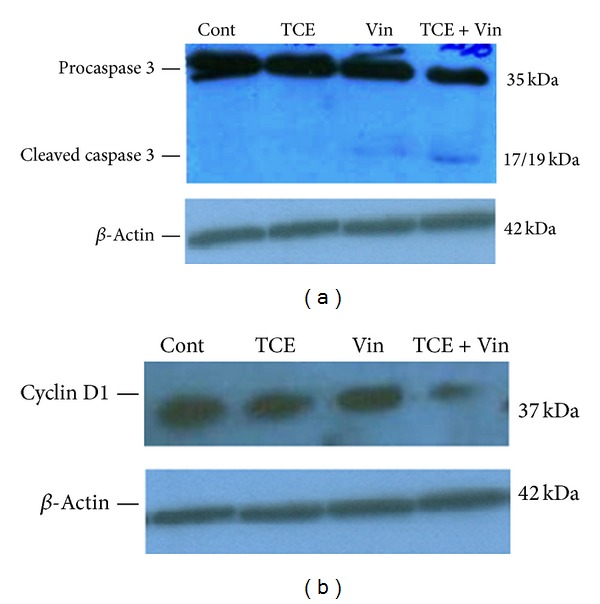
Effect of subeffective doses of* T. caramanicus* extract (TCE, 40 *μ*g/mL) and vincristine (VIN, 150 nM) alone or in combination on the levels of cleaved caspase 3 (a) and cyclin D1 (b) in MCF-7 cancer cells. *β*-actin was used as an internal control for loading.

To examine the synergic effect of TCE and vincristine on the upregulation of activated caspase 3 and downregulation of cyclin D1, the cells were exposed to control medium, low effective dose of TCE (40 *μ*g/mL), and vincristine alone or in combination for 24 h. The blots showed that TCE and vincristine cotreatment markedly increased cleaved caspase 3 (Figure 6(a)) and decreased Cyclin D1 protein expression (Figure 6(b)) in MCF-7 cells.

## 4. Discussion

Cancer is a very complex disease and the occurrence and development of tumor cells are closely related to abnormal intracellular signal transduction system [10]. Nowadays, one of the main methods of modern cancer treatment is chemotherapy. However, most chemotherapeutic agents have different substantial short- and long-term side effects. Thus, in recent years, major research has been focused on herbs and plants which have been considered for being nontoxic and for the prevention and treatment of certain types of cancer.

In the present study, the extract of* T. caramanicus* could decrease cell viability of MCF-7 breast cancer cell line. TCE-induced cell toxicity is performed through the induction of apoptosis and inhibition of cell proliferation (Figures 3, 4, and 5).

The origin of cancer involves the exaggerated cellular proliferation, as well as the suppression of apoptotic processes. Apoptosis is an important type of cell death in response to cytotoxic candidate in cancerous situation. It has been documented that many natural compounds with anticancer property could induce apoptosis of tumor cells [11]. In addition, numerous scientific reports have demonstrated that the induction of apoptotic death in cancer therapy is strikingly associated with activation of caspase 3 [12]. Since caspase 3 has a central role in apoptosis and is supposed to be the final executor of apoptosis pathway we evaluated the effect of TCE on activated caspase 3 level in MCF-7 cells.

In addition to receptor-mediated apoptosis, there is another pathway activated by cytotoxic compounds. It occurs by alteration in mitochondrial permeability and subsequent cytochrome c release and formation of the apoptosome and activation of caspase 9 and then caspase 3 resulting in downstream events involved in cell death. It has been revealed that the release of cytochrome c is regulated by Bcl2 family proteins. Antiapoptotic Bcl2 family members exist in the outer mitochondrial membrane and prevent cytochrome c release, while proapoptotic members are translocated to the mitochondria to induce apoptosis either by forming pores in mitochondria directly or by antagonizing the antiapoptotic proteins [13].

Cyclin D1, a subunit of CDK_4_ and CDK_6_, is one of the major biochemical switches in cell cycle [14]. Upregulation of this functional protein is also observed in several malignancies, including breast, prostate, neck, and head cancers [15–17]. It has been documented that some phytochemicals, like curcumin, resveratrol, genistein, and apigenin, can reduce cyclin D1 overexpression in cancer cells [18–21].

Surprisingly, the data showed that TCE significantly promotes cell damage, activates caspase 3, and elevates Bax/Bcl2 ratio. In addition, the expression of Cyclin D1 significantly reduces in TCE-treated MCF-7 cells. Therefore, this plant can be introduced as a candidate for more study in cancer therapy.

*T. caramanicus* has polyphenols that may be responsible for its observed anticancer effect in this study. Carvacrol (51.0%) and thymol (20.84%) are the most active components of* T. caramanicus*. It has been shown that carvacrol has antiproliferative properties on non-small-cell lung cancer cells, A549, chronic myeloid leukemia cells, K562, Hep-2 cells, murine B16 melanoma cells, and human metastatic breast cancer cells, MDA-MB231 [22, 23]. In addition, the anticancer properties of thymol have also been reported in cancer cells [24].

Recently, it has been reported that the anticancer effects of carvacrol in metastatic breast cancer cells (MDA-MB231) were based on the activation of the classical apoptosis response, including decrease in mitochondrial membrane potential and increase in cytochrome c release from mitochondria, decrease in Bcl-2/Bax ratio, increase in caspase activity, and cleavage of PARP and fragmentation of DNA, which belong to the mitochondrial pathway of the apoptosis [25].

Furthermore, antioxidant properties of thyme extracts have been reported in numerous papers [8, 26, 27]. Natural antioxidants with their ability to scavenge free radicals can protect the cells from different diseases such as cancer [28].

Numerous* in vitro *studies demonstrated that cancer cells may develop resistance to chemotherapeutic agents and lead to the development of a phenotype exhibiting multidrug resistance [29]. Upregulation of ATP binding cassette transporters, such as P-glycoprotein (P-gp), multidrug resistance associated protein, and breast cancer resistance protein, are the most well-known mechanisms of drug resistance. Furthermore, downregulation of the caspase cascade is another documented drug resistance mechanism so that resistance to apoptosis is correlated with reduced caspase 3 activity in some cancer cell lines [30].

Recently, a phase-specific chemotherapy resistance due to epidermal growth factor receptor (EGFR) has been demonstrated in human breast cancer cells. Surprisingly, cyclin D1 is involved in such EGFR-mediated multidrug resistance [31, 32].

As mentioned above, multidrug resistance is a major obstacle to successful chemotherapy for breast cancer and finding novel resistance reversers for enhancing drugs power and reducing their doses are critically needed. In the present study, we evaluated whether thyme extract could enhance vincristine-induced MCF-7 toxicity. The data showed that TCE potentiates the cytotoxicity of vincristine which is accompanied by an increase in cleaved caspase 3 and a decrease in Cyclin D1 protein expression (Figure 5). However, the detailed mechanisms involved in this interaction between vincristine and thyme extract remain unclear and should be clarified by further investigation.

## 5. Conclusion

Taken together, this study indicates that* T. caramanicus* extract has a potential antiproliferative/proapoptotic property in MCF-7 cells and can be used as pharmaceutic case study for breast cancer treatments.

## References

[1] Siegel R, Naishadham D, Jemal A (2012). Cancer statistics, 2012. *CA Cancer Journal for Clinicians*.

[2] Hernandez-Aya LF, Gonzalez-Angulo AM (2013). Adjuvant systemic therapies in breast cancer. *Surgical Clinics for North America*.

[3] Lee KW, Bode AM, Dong Z (2011). Molecular targets of phytochemicals for cancer prevention. *Nature Reviews Cancer*.

[4] Fahey JW, Talalay P, Kensler TW (2012). Notes from the field: “green” chemoprevention as frugal medicine. *Cancer Prevention Research*.

[5] Hudaib M, Speroni E, Di Pietra AM, Cavrini V (2002). GC/MS evaluation of thyme (*Thymus vulgaris* L.) oil composition and variations during the vegetative cycle. *Journal of Pharmaceutical and Biomedical Analysis*.

[6] Bauer KD, Garbe H, Surburg H (1997). *Common Fragrance and Flavor Materials*.

[7] Amiri H (2012). Essential oils composition and antioxidant properties of three thymus species. *Evidence-Based Complementary and Alternative Medicine*.

[8] Nejad Ebrahimi S, Hadian J, Mirjalili MH, Sonboli A, Yousefzadi M (2008). Essential oil composition and antibacterial activity of Thymus caramanicus at different phenological stages. *Food Chemistry*.

[9] Safaei-Ghomi J, Ebrahimabadi AH, Djafari-Bidgoli Z, Batooli H (2009). GC/MS analysis and in vitro antioxidant activity of essential oil and methanol extracts of Thymus caramanicus Jalas and its main constituent carvacrol. *Food Chemistry*.

[10] Wolf B, Brischwein M, Lob V, Ressler J, Wiest J (2007). Cellular signaling: aspects for tumor diagnosis and therapy. *Biomedizinische Technik*.

[11] Lu JJ, Dang YY, Huang M, Xu WS, Chen XP, Wang YT (2012). Anti-cancer properties of terpenoids isolated from Rhizoma Curcumae—a review. *Journal of Ethnopharmacology*.

[12] Hunter AM, LaCasse EC, Korneluk RG (2007). The inhibitors of apoptosis (IAPs) as cancer targets. *Apoptosis*.

[13] Wu CC, Bratton SB (2013). Regulation of the intrinsic apoptosis pathway by reactive oxygen species. *Antioxidants and Redox Signaling*.

[14] Baldin V, Lukas J, Marcote MJ, Pagano M, Draetta G (1993). Cyclin D1 is a nuclear protein required for cell cycle progression in G1. *Genes and Development*.

[15] Adelaide J, Monges G, Derderian C, Seitz J-F, Birnbaum D (1995). Oesophageal cancer and amplification of the human cyclin D gene CCND1/PRAD1. *British Journal of Cancer*.

[16] Gumbiner LM, Gumerlock PH, Mack PC (1999). Overexpression of cyclin D1 is rare in human prostate carcinoma. *Prostate*.

[17] Peurala E, Koivunen P, Haapasaari K-M, Bloigu R, Jukkola-Vuorinen A (2013). The prognostic significance and value of cyclin D1, CDK4 and p16 in human breast cancer. *Breast Cancer Research*.

[18] Aggarwal BB, Bhardwaj A, Aggarwal RS, Seeram NP, Shishodia S, Takada Y (2004). Role of resveratrol in prevention and therapy of cancer: preclinical and clinical studies. *Anticancer Research*.

[19] Shukla S, Gupta S (2007). Apigenin-induced cell cycle arrest is mediated by modulation of MAPK, PI3K-Akt, and loss of cyclin D1 associated retinoblastoma dephosphorylation in human prostate cancer cells. *Cell Cycle*.

[20] Shehzad A, Wahid F, Lee YS (2010). Curcumin in cancer chemoprevention: molecular targets, pharmacokinetics, bioavailability, and clinical trials. *Archiv der Pharmazie*.

[21] Hwang KA, Kang NH, Yi BR, Lee HR, Park MA, Choi KC (2013). Genistein, a soyphytoestrogen, prevents the growth of BG-1 ovarian cancer cells induced by 17*β*-estradiol or bisphenol A via the inhibition of cell cycle progression. *International Journal of Oncology*.

[22] Koparal AT, Zeytinoglu M (2003). Effects of carvacrol on a human non-small cell lung cancer (NSCLC) cell line, A549. *Cytotechnology*.

[23] Arunasree KM (2010). Anti-proliferative effects of carvacrol on a human metastatic breast cancer cell line, MDA-MB 231. *Phytomedicine*.

[24] Satooka H, Kubo I (2012). Effects of thymol on B16-F10 melanoma cells. *Journal of Agricultural and Food Chemistry*.

[25] Karkabounas S, Kostoula OK, Daskalou T (2006). Anticarcinogenic and antiplatelet effects of carvacrol. *Experimental Oncology*.

[26] Miura K, Kikuzaki H, Nakatani N (2002). Antioxidant activity of chemical components from sage (*Salvia officinalis* L.) and thyme (*Thymus vulgaris* L.) measured by the oil stability index method. *Journal of Agricultural and Food Chemistry*.

[27] Cerda A, Martínez ME, Soto C (2013). The enhancement of antioxidant compounds extracted from Thymus vulgaris using enzymes and the effect of extracting solven. *Food Chemistry*.

[28] Niture SK, Rao US, Srivenugopal KS (2006). Chemopreventative strategies targeting the MGMT repair protein: augmented expression in human lymphocytes and tumor cells by ethanolic and aqueous extracts of several Indian medicinal plants. *International Journal of Oncology*.

[29] Kars MD, Işeri ÖD, Gündüz U, Ural AU, Arpaci F, Molnár J (2006). Development of rational *in vitro* models for drug resistance in breast cancer and modulation of MDR by selected compounds. *Anticancer Research*.

[30] Ding Z, Yang X, Pater A, Tang S-C (2000). Resistance to apoptosis is correlated with the reduced caspase-3 activation and enhanced expression of antiapoptotic proteins in human cervical multidrug-resistant cells. *Biochemical and Biophysical Research Communications*.

[31] Xu J-W, Li Q-Q, Tao L-L (2011). Involvement of EGFR in the promotion of malignant properties in multidrug resistant breast cancer cells. *International Journal of Oncology*.

[32] Chen S-J, Luan J, Zhang H-S (2012). EGFR-mediated G1/S transition contributes to the multidrug resistance in breast cancer cells. *Molecular Biology Reports*.

